# Pattern of Maternal Knowledge and Its Implications for Diarrhoea Control in Southern Malawi: Multilevel Thresholds of Change Analysis

**DOI:** 10.3390/ijerph9030955

**Published:** 2012-03-16

**Authors:** Salule Joseph Masangwi, Anthony Martin Grimason, Tracy Dawn Morse, Lawrence Kazembe, Neil Ferguson, George Christopher Jabu

**Affiliations:** 1 Centre for Water, Sanitation, Health and Appropriate Technology Development (WASHTED), University of Malawi, The Polytechnic, P/B 303, Blantyre, Malawi; 2 Environmental Health, Department of Civil Engineering, University of Strathclyde, Glasgow, G4 0NG, UK; Email: a.m.grimason@strath.ac.uk (A.M.G.); tracythomson@africa-online.net (T.D.M.); n.s.ferguson@strath.ac.uk (N.F.); 3 Department of Mathematics and Statistics, University of Malawi, The Polytechnic, P/B 303, Blantyre 3, Malawi; 4 Scotland Chikhwawa Health Initiative (SCHI), P.O. Box 30376, Blantyre 3, Malawi; 5 Africa Academy for Environmental Health, P.O. Box 15574, Sinoville 0129, South Africa; 6 Environmental Health Department, University of Malawi, P/B 303, Chichiri, Blantyre 3, Malawi; Email: gjabu@poly.ac.mw; 7 Department of Mathematical Sciences, University of Malawi, Chancellor College, P.O. Box 280, Zomba, Malawi

**Keywords:** responsible mother’s knowledge, pattern of variation, diarrhoea control, multilevel threshold of change, southern-tip of Malawi

## Abstract

A survey was conducted in Southern Malawi to examine the pattern of mothers’ knowledge on diarrhoea. Diarrhoea morbidity in the district is estimated at 24.4%, statistically higher than the national average at 17%. Using hierarchically built data from a survey, a multilevel threshold of change analysis was used to determine predictors of knowledge about diarrhoeal aetiology, clinical features, and prevention. The results show a strong hierarchical structured pattern in overall maternal knowledge revealing differences between communities. Responsible mothers with primary or secondary school education were more likely to give more correct answers on diarrhoea knowledge than those without any formal education. Responsible mothers from communities without a health surveillance assistant were less likely to give more correct answers. The results show that differences in diarrhoeal knowledge do exist between communities and demonstrate that basic formal education is important in responsible mother’s understanding of diseases. The results also reveal the positive impact health surveillance assistants have in rural communities.

## 1. Introduction

The importance of safe water sources, improved sanitation and good hygiene practices in controlling diarrhoeal diseases is well documented. For example, a World Bank Report prepared in 2004 observed that safe water supply, water quality, sanitation, hygiene or multi-factorial interventions have significantly reduced diarrhoea morbidity globally [1]. Another study carried out in Malawi [2] showed that water point-of-use treatment, household connections without household storage, hand-washing, and sanitation contributed to the decrease of diarrhoea. In East Africa, Tumwine *et al*. [3] observed that poor hygiene, especially unsafe disposal of faeces and waste water, obtaining water from surface sources or wells were some of the factors that cause diarrhoea morbidity. 

Although diarrhoea can be reduced through improvements in the above-stated areas it has been shown that success and effectiveness of intervention programmes depend on the participation, behaviour and reaction of households in the communities [4,5]. However, the level of understanding in rural communities of developing countries including Malawi on the use of safe water, improved sanitation and good hygiene practices appears to be limited. More worrying is the fact that some health education providers such as health surveillance assistants (HSAs) have limited education, limited occupational training, and limited knowledge of diarrhoea and its control measures [2]. 

This study examines the pattern of matriarchal figures’ knowledge about the aetiology, clinical features, and prevention of diarrhoea in Chikhwawa, Malawi. It further explores the impact of various determining factors including HSAs, village health committees (VHCs), and NGOs on household knowledge through responses from a matriarchal figure in each household. Apart from being custodians of household information, matriarchal figures’ knowledge and hygiene practices especially in handling drinking water, food, and children’s faecal matter can affect diarrhoea transmission [6,7]. Their knowledge in this study was, therefore, considered of paramount importance. 

## 2. Methods

### 2.1. Area

A survey was conducted in Chikhwawa, a district in Southern Malawi, to obtain a representative sample of individuals, households, and villages. Chikhwawa has a surface area of 4755 km^2^ with an elevation of only 100 m above sea level. Out of a population of approximately 477,524 people in the district, seventeen percent are children under five years of age, twenty three percent are women of child bearing age, and the estimated number of expectant women is 23,876 [8]. Chikhwawa is faced with a number of environmental and socioeconomic problems that are responsible for various infectious diseases. Currently diarrhoea morbidity in the district is estimated at 24.4% [8,9]. This is statistically higher than the national average of 17% [10]. 

### 2.2. Sample

A two-stage survey methodology was adopted to produce a district representative sample of households. The first-stage involved sampling of villages that were strategically selected with a probability proportional to the number of enumeration areas in each traditional authority (Chikhwawa has eleven traditional authorities and each traditional authority has several villages under its jurisdiction). The second sampling stage took place on the day of interviews. Households were systematically chosen with equal probability sampling. Only responsible mothers in each household were eligible for interviews and all other members of the households were asked to leave the interviewing premises to avoid interference. A “responsible mother” in this study is a woman who is the owner or the most senior responsible woman in a household. 

Ten enumerators were recruited amongst those that had already been involved in national surveys at the National Statistical Office in Malawi. These were given one week intensive training on the questionnaire and had two days of a pilot study in order to: (a) acquaint themselves with the questions; (b) afford them an opportunity to ask questions, seek clarifications, and make general questions where necessary; (c) accustom them with survey, interviewing, and house selection techniques. The exercise also sought to familiarise them with the problems they were going to encounter in the field and at the same time share their previous experiences in such exercises. Since the questionnaire was translated in a local language, the pilot study was also meant to clear any ambiguities and problems in its interpretation and administration

Only responsible mothers for upkeep of the household were interviewed. Household sizes ranged from 1 to 13. After discarding missing and uncompleted data information for a total number of 1389 households nested within 33 villages were used in this study. 

### 2.3. Variables

#### 2.3.1. Outcome Variables

Distribution of outcome variables is listed in Table 1. A responsible mother in each household was asked to mention: (i) the symptoms of; (ii) causes of; and (iii) prevention methods against diarrhoea. These questions were open ended to reduce the risk of bias. A series of binary responses were formed by coding 1 if a responsible mother mentioned a required symptom, prevention method, or cause and 0 otherwise. Categories of responses for each outcome were formed. Three categories were formed for the symptoms category and these are: (1) zero or one symptom mentioned; (2) two symptoms mentioned; and (3) three or more symptoms mentioned. Four categories each were formed for prevention method and causes of diarrhoea as follows: (1) no single cause or no single prevention method mentioned; (2) one cause or one preventative method mentioned; (3) two causes or two prevention methods mentioned; and (4) three or more causes or three or more prevention methods mentioned.

**Table 1:** Descriptive statistics for response variables on diarrhoea knowledge.

Variable	*Mean*	*Median*	*Min.*	*Max.*	*N* = 1389	%
*What are the symptoms of diarrhoea?*						
1. Watery stools					1,171	84.3
2. Increased number of stools					183	13.2
3. Loose stools					383	27.6
4. Loose stools and vomiting					175	12.6
5. Bloody stools					183	13.2
6. Stomach-ache					492	35.4
*What are the causes of diarrhoea*						
1. Contaminated water					765	55.1
2. Contaminated food					619	44.6
3. Flies					376	27.1
4. Poor hygiene and sanitation practices					703	50.6
5. Poor sanitation practices					*111*	*8.0*
*What action do you take to prevent diarrhoea?*						
1. Add disinfectant (water guard, chlorine, *etc*.) to water					421	30.3
2. Good water hygiene or management					357	25.7
3. Good food hygiene or management					78	5.6
4. Proper cleaning of cooking and eating utensils					332	23.9
5. Good sanitation					278	20.0
6. Hands washing					307	22.1
*Number of Symptoms identified*	*2*	*2*	*0*	*5*		
Zero or one symptom					351	25.3
Two symptoms					672	48.4
Three or more symptoms					366	26.3
*Number of Causes identified*	*2*	*2*	*0*	*5*		
No single cause mentioned					61	4.4
One cause mentioned					590	42.5
Two causes mentioned					365	26.3
Three or more causes mentioned					373	26.9
*Prevention methods identified*	*1*	*1*	*0*	*6*		
No prevention method					435	31.3
One prevention method					404	29.1
Two prevention methods					338	24.3
Three or more methods					212	15.3
*Overall knowledge*	*5*	*5*	*0*	*14*		
Zero to three points					305	22.0
Four to five points					498	35.9
Six to seven points					438	31.5
Eight or more points					148	10.7

A fourth response variable was formed by taking the total scores for each responsible mother in all the three response categories. This outcome variable was called the overall knowledge on diarrhoea. Its categories are: (1) zero to three points scored; (2) four to five points scored; (3) six to seven points scored; (4) eight or more points scored. Note that the cut-off points for the categories are arbitrary and are guided only by the distribution of mothers in the knowledge profile. 

In Malawian vernacular language diarrhoea is known as “kutsegula m’mimba” literally meaning opening up of bowels which is associated with defecation of watery stools, especially in young children. However “bloody stools” or dysentery, are known as “kamwazi” assumed to be a different disease from diarrhoea. Interviewers were, therefore, advised to use both vernacular definitions of diarrhoea and dysentery when collecting data on diarrhoeal illness

Since the category scores on maternal knowledge are arbitrary, ordered and hierarchical, in order to retain category values, determine the extent to which each predictor variable contributes to maternal knowledge, and account for the hierarchical structure the analysis was performed using multilevel thresholds of change. Notice that the number of responses given to each question on outcomes is meant to test the extent of knowledge and flexibility in expressing such knowledge. 

#### 2.3.2. Explanatory Variables Included in the Models

Only variables that were significant at *p* = *0.20* when tested with the outcome variables were included in the final model. The following explanatory variables were included: Responsible mother’s age, highest level of responsible mother’s schooling, relative wealth, health facility, availability of health surveillance assistants (HSAs) and existence of an non-governmental organisations (NGOs). 

Malawi’s health service delivery system is consisted of four levels: community, primary, secondary, and tertiary care levels. Community level focuses on preventive interventions and service is mainly provided through HSAs and VHCs. Primary care is delivered through health centres while secondary and tertiary care services are provided through district and central hospitals. Government is the main provider of health services in Malawi with approximately 60% of all services. Christian Association of Malawi (CHAM), faith based organisations, are responsible for the provision of about 37% of all services. Other providers include NGOs (both private-for profit and private not-for-profit). 

### 2.4. Statistical Analysis and Estimation

The ordered multinomial response model [11,12,13,14,15] is used to explain the probability of ordered scores on diarrhoea knowledge. The response variable is the number of answers correctly given by each responsible mother in a household on symptoms, causes or prevention of diarrhoea. The response on symptoms has three categories 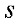 such that *s* = 1, 2, 3. The responses on causes, prevention and overall knowledge have four categories 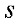 each such that *s* = 1, 2, 3, 4. The last category in each case is taken as a reference. 

Suppose that the probability of a woman from household *i* in community *j* of having a score in category *s* is 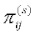 and the probability that household *i* in community *j* will obtain a score higher than that represented by category *s* is 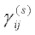. Then the cumulative response probabilities are defined as: 


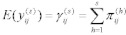
 (1)

where *s* = 1, 2 on the symptoms outcome and *s* = 1, 2, 3 on causes, prevention and overall knowledge outcomes. For the symptoms response variable 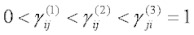 and for the other response variables 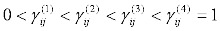. Notice that the probabilities for the scores are cumulated downwards for convenience in interpretation of the results. A proportional odds model with a 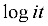 link is, therefore, given by:


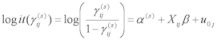
 (2)

with *α*^s^ as threshold values for the scores. *X_ij_* is the covariate vector and *β* is a vector of unknown fixed regression parameters. Also, *u*_0*j*_ is a design vector of random effects. Fixed and random effects operate linearly on thresholds and hence indirectly on the probabilities over the ordered scores. For the *j* thcommunity establishment there is a single random effect *u*_0*j*_ which is normally distributed with mean 0 and variance 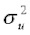.

In our model (2) we have assumed that fixed cut-point thresholds do not vary across observations. However, if tests of parallel lines for different predictor variables on their respective outcomes show that some slope coefficients are not the same across response categories, then model (2) can be extended to accommodate this scenario [11]. Hence, Equation 2 is rewritten as:


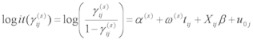
 (3)

where 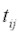 is a predictor variable whose slope coefficients, 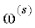, are not the same across response categories and hence allowing fixed cut-point thresholds to vary across observations. Thus:


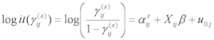


with: 


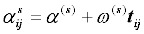



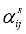
 is now our threshold value while 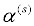 is defined as a baseline threshold. Model (3) which includes multilevel random effects is known as the multilevel thresholds of change model (MTCM) [11]

Each predictor variable was tested for a test of parallel lines with each of the outcome variables included in this study. All predictor variables that satisfied the test *i.e.*, if *p ≥ 0.20* for a given outcome variable were included in the proportional odds model. All those that did not satisfy the test *i.e.*, if *p* < 0.2 were allowed to vary across different cut-off points, thus contributed to the baseline threshold point of different categories. 

Estimation of the parameters was performed using Markov Chain Monte Carlo (MCMC) procedures in MLwiN 2.10 software [16]. MCMC procedures are used to avoid underestimation of random effects which may occur if marginal and penalized quasi-likelihood estimation methods are used in MLwiN [17]. However, initial estimates to obtainprior samples for the procedures were derived using second-order penalised quasi-likelihood (PQL) procedures with Iterative generalised least-squares (IGLS) estimation. Stability of all model parameters in MCMC procedures was monitored by observing the Raftery-Lewis diagnostics [16]. The maximum number of iterations performed was 70,000.

## 3. Results

Distribution of response variables is summarised in Table 1. Almost all of the responsible mothers interviewed mentioned watery stools as the likely symptom of diarrhoea. Very few responsible mothers mentioned additional symptoms such as increased number of stools (13%), loose stools (28%), loose stools and vomiting (13%), bloody stools (13%), and stomach-ache (35%). One in every two responsible mothers mentioned contaminated water as a means through which one can contract diarrhoea. Similarly one in every two responsible mothers mentioned contaminated food and poor hygiene practices as a cause of diarrhoea. One in four (27%) mentioned flies while one in twelve (8%) mentioned poor sanitation practices as pathways through which people can get diarrhoea. Few responsible mothers knew how diarrhoea can be prevented. One in three (30%) responsible mothers mentioned the use of water disinfectants, while one in every four responsible mothers mentioned good water hygiene and management. One responsible mother in every four mentioned proper cleaning of cooking and eating utensils. Good sanitation was mentioned by one in five (20%) women, hands washing by a similar ratio (22%), and good food hygiene or management by approximately one in seventeen women (6%). In general two thirds of the responsible mothers interviewed mentioned at most one diarrhoea symptom, half mentioned at most one cause of diarrhoea, and two thirds of responsible mothers mentioned at most one preventative method. Table 2 shows descriptive statistics for predictor variables used in the models. Most households sought care from either a government hospital (30%) or a health centre (48%). One in every seven households (14%) sought care from a Christian Association of Malawi (CHAM) hospital, one in every 50 households (2%) sought care from local health posts, and one in every 14 households (7%) sought care from private clinics. Almost half the households were in communities without an NGO and three in every 10 households lived in communities without an HSA.

**Table 2:** Descriptive statistics for predictor variables on diarrhoea knowledge.

Variable	*Mean*	*Median*	*Min.*	*Max.*	*N* = 1389	%
*Mother attended School*						
1. None					290	20.9
2. Primary					812	58.5
3. At least secondary					287	20.7
*Type of Health Facility*						
1. Government hospital					417	30.0
2. Government health centre					672	48.4
3. Christian Association of Malawi (CHAM)					189	13.6
4. Local health post					21	1.5
5. Local private clinic					90	6.5
*Whether Non Governmental Organisation(NGO)*						
1. Exists					611	44.0
2. Does not exist					778	56.0
*Whether Health Surveillance Assistant (HSA)*							
1. Exists					951	68.5	
2. Does not exist					438	31.5	
*Maternal age*	*35*	*30*	*15*	*89*	*1,389*	*100*	

MTCM used to identify predictors of responsible mother’s knowledge on diarrhoea symptoms is given in Table 3. The baseline threshold parameters are significantly different from zero and, therefore, consistently contributed to the score values of probability in the different categories of the diarrhoea symptom outcome variable. Primary school education decreased the responsible mother’s threshold probability of mentioning one or no diarrhoea symptom when compared to responsible mothers without any formal school education *i.e.*, primary education increased responsible mother’s probability of mentioning two or more diarrhoea symptoms when compared to those without any formal education. Secondary education decreased responsible mother’s threshold probability of mentioning at most one symptom and of mentioning two symptoms i.e., secondary education increased responsible mother’s likelihood of mentioning more than two diarrhoea symptoms. 

**Table 3:** Partial proportional odds models to identify determinants of responsible mothers’ knowledge on diarrhoea symptoms.

Variable	Estimate	95% CI
**Threshold**		
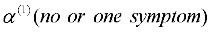	−1.465	(−1.986,−0.944)
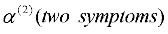	1.053	(0.508,1.598)
**No School**	**(Reference group)**	
**Primary school**		
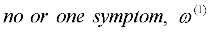	−0.233	(−0.572,0.106)
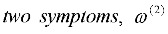	−0.572	(−0.935,−0.209)
**Secondary School**		
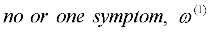	−0.364	(−0.795,0.067)
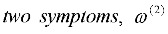	−0.534	(−0.975,−0.093)
**Government hospital**	**(Reference group)**	
**Government Health centre**		
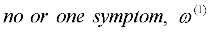	0.238	(−0.148,0.624)
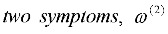	0.323	(−0.055,0.701)
**CHAM**		
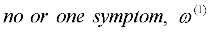	0.684	(0.092,1.276)
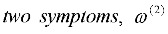	1.331	(0.725,1.937)
**Health Post**		
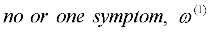	1.023	(0.049,1.997)
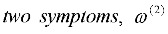	1.307	(−0.081,2.695)
**Local private clinic**		
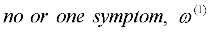	−0.063	(−0.806,0.680)
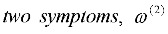	−0.170	(−0.781,0.441)
**Age**		
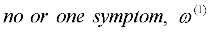	−0.306	(−0.453,−0.159)
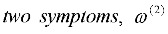	0.028	(−0.113,0.169)
**NGO exists**	**(Reference group)**	
NGO does not exist 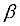	0.275	(−0.011,0.561)
**HSA exists**	**(Reference group)**	
HSA does not exist 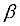	−0.091	(−0.328,0.146)
Community effects 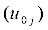	0.623	(0.219,1.027)

CI = Credible Interval.

Threshold probability of mentioning two symptoms increased for responsible mothers that sought care from a health centre when compared to those that sought care from a government hospital. However, thresholds increased for women who mentioned two symptoms and those who mentioned at most one symptom if their health facility was either a CHAM hospital or a health post. This means probabilities of a responsible mother mentioning more than two symptoms decreased if their health facility was either CHAM or a health post. 

Increasing responsible mother’s age decreased the threshold probability of obtaining one or no diarrhoea symptom *i.e.*, increasing responsible mother’s age increased chances of mentioning more than one diarrhoea symptom.

Existence of an NGO was the only significant predictor variable that obeyed proportional odds features. Responsible mothers from communities without an NGO were more likely to mention fewer symptoms than those whose communities have the services of NGOs.

Table 4 shows partial proportional odds models to identify predictors of diarrhoea causes, prevention, and overall knowledge. All baseline thresholds for causes and prevention methods outcomes are significantly different from zero and, therefore, substantially contribute to the score values of their outcome probability in different categories. Thresholds probabilities for those that mention no single cause, one cause, or two causes of diarrhoea significantly increases for communities without HSAs relative to those with HSAs and respectively]. Nearest health facility and responsible mother’s age were the only significant predictors that obeyed proportional odds features. 

**Table 4:** Partial proportional odds models to identify determinants of responsible mothers’ knowledge on overall knowledge, causes and prevention of diarrhoea.

Variable	Causes of diarrhoea		Prevention methods		Overall knowledge on diarrhoea	
	estimate	95% CI	estimate	95% CI	estimate	95% CI
Threshold 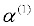	−4.122	(−4.782,−3.461)	−0.803	(−1.160,−0.446)	−1.653	(−2.235,−1.071)
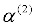	−0.756	(−1.211,−0.301)	0.737	(0.380,1.094)	−0.137	(−0.691,0.418)
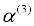	0.615	(0.158,1.072)	2.209	(1.750,2.668)	1.281	(0.601,1.961)
Primary school 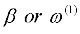	−0.069	(−0.339,0.201)	−0.090	(−0.349,0.169)	−0.289	(−0.554,−0.024)
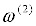						
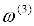						
Secondary School 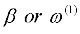	0.070	(−0.275,0.415)	−0.415	(−0.740,−0.090)	−0.418	(−0.757,−0.079)
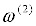						
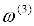						
Health centre 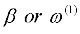	0.625	(0.307,0.942)	−0.170	(−0.491,0.151)	0.199	(−0.118,0.516)
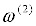			−0.583	(−0.902,−0.263)		
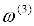			−0.357	(−0.764,0.051)		
CHAM 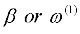	1.044	(0.518,1.569)	0.220	(−0.237,0.677)	0.940	(0.436,1.444)
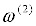			−0.211	(−0.652,0.230)		
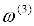			−0.053	(−0.610,0.504)		
Health Post 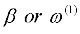	−0.244	(−1.085,0.597)	−0.092	(−1.127,0.943)	0.054	(−0.765,0.873)
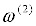			−0.716	(−1.633,0.201)		
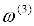			−0.019	(−1.366,1.327)		
Local private clinic 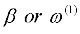	0.844	(0.276,1.412)	0.247	(−0.312,0.806)	0.442	(−0.099,0.983)
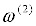			0.127	(−0.412,0.666)		
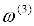			0.150	(−0.558,0.858)		
Age 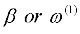	0.126	(0.020,0.232)	0.180	(0.074,0.286)	0.002	(−0.008,0.012)
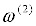					0.008	(0.000,0.016)
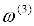					0.025	(0.009,0.041)
No NGO 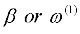	−0.030	(−0.297,0.237)	0.278	(0.002,0.554)	0.207	(−0.056,0.470)
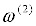			0.142	(−0.117,0.401)		
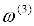			−0.007	(−0.336,0.322)		
No HSA 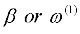	1.040	(0.507,1.573)	0.617	(0.362,0.872)	0.605	(0.380,0.830)
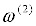	0.571	(0.320,0.822)	0.466	(0.217,0.715)		
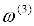	0.611	(0.323,0.899)	0.578	(0.225,0.931)		
Community effects ( 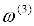 )	0.352	(0.085,0.619)	0.063	(−0.027,0.153)	0.366	(0.107,0.625)

Respondents whose health facility was a health centre, a CHAM hospital, or a local private clinic were likely to mention fewer causes of diarrhoea than those whose health facility is a government hospital. The older the responsible mother the more likely they were to mention fewer causes of diarrhoea. 

Thresholds probabilities for those that mention no single prevention method, one prevention method or two prevention methods decrease for households nearest to health centres than those nearest government hospitals. However, threshold probabilities for those that mention no single prevention method, one prevention method or two prevention methods increase for households that do not have HSAs in their communities. 

School level and responsible mother’s age were the only significant indicators that obeyed proportional odds features in the prevention methods outcome. Responsible mothers with at least secondary school education were less likely to mention fewer prevention methods when compared to those who had not attended any formal school and older responsible mothers were more likely to mention fewer prevention methods. 

On overall knowledge, baseline threshold probability of having scored a total of four to five or six to seven points increased with responsible mother’s age. School level, nearest health facility, and existence of an HSA were significant predictors that obeyed proportional odds features in the overall knowledge outcome. Responsible mothers that attended primary school and those that attended at least secondary school were less likely to score fewer points than those who did not attend any formal school education. Households whose nearest health facility was a CHAM hospital were more likely to score fewer points than those near a government hospital. Responsible mothers in communities without HSAs were more likely to score fewer points than those in areas that have HSAs. 

**Figure 1:**
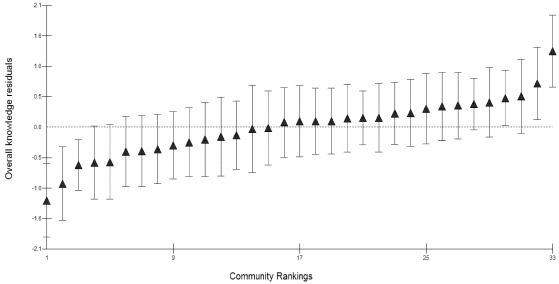
Catapillar plot of diarrhoea knowledge residuals ranked by sampled communities. The dotted line is the mean of the estimated (shrunken) residuals * which is equal to zero. The triangles indicate estimated (shrunken) community residuals. * Estimated or shrunken residual for community *j* is the residual obtained by multiplying the mean of the residuals of households in community *j* by a shrinkage factor. Shrinkage factor shrinks an observed group mean towards the centre of the population mean.

There is significant variation between communities on the symptoms outcome, causes outcome, and overall knowledge outcome indicating differences between communities in those outcomes. This is evident in Figure 1 which shows residuals for some communities significantly below zero while others are significantly above zero revealing disparities in the way different communities understand issues related to diarrhoea. 

## 4. Discussion

This study was undertaken to examine community variation and factors associated with responsible mother’s knowledge on causes of diarrhoeal illness, its symptoms and how to prevent the disease in rural villages in Chikhwawa, Malawi. The majority of responsible mothers in Chikhwawa (84%) used the definition “watery stools” to identify diarrhoea. Few responsible mothers mentioned “increased number of stools” or “bloody stools”. Approximately one on three mentioned “stomach-ache”. These results may be a consequence of a local Malawian definition of diarrhoea, “*kutsegula m’mimba*” which literally means opening up of bowels associated with defecation of watery stools especially in young children. The vernacular definition does not include the words “increased” or “bloody” stools. To avoid under reporting of causes and preventative measures the interviewers were advised to use both definitions of diarrhoea when seeking responses from responsible mothers in the communities. 

About a quarter or less of the responsible mothers mentioned each of the preventive measures while only half managed to mention half of the causes of diarrhoea. Clearly the results show inadequate knowledge amongst responsible mothers both on causes and preventative measures against diarrhoea. While many responsible mothers were able to identify diarrhoea (as watery stools) only a few could link diarrhoea to risks that go with it and many more were unaware of the actions they can take to avoid contracting the disease. The responses may be a reflection of health education awareness messages availed to mothers in the communities or lack thereof as is evident in the significant relationship between the location of the health facility and knowledge. Similarly the significant relationship between presence of an HSA within the village and knowledge explains the important role HSAs are playing in preventive health in the rural communities. As already explained in the methods section, HSAs are resident within communities where they undertake and assist in the operation of health posts or clinics, outreach clinics and health surveillance within villages. They also carry out all water and sanitation development and regular village inspections to determine if acceptable standards of living are being met. They are also responsible as health educators [2]. Taking into consideration the results of this MTCM analysis, it is apparent that communities without an HSA or VHCs are less knowledgeable about the causes, symptoms, and prevention of diarrhoeal disease and they miss the services from HSAs and VHCs. 

Responsible mother’s age and education were found to be significant predictors of responsible mothers’ knowledge on diarrhoea symptoms. Education increased responsible mothers’ ability to mention more diarrhoea symptoms and preventative measures. It also increased chances of more overall knowledge on diarrhoea. Other earlier studies in Malawi [18], Laos People’s Democratic Republic [19], and the Colombian Pacific [20] agree with this finding and observed that higher literacy levels were positively related to knowledge on diarrhoea or malaria.

There are mixed results on responsible mother’s age and knowledge. There is a positive relationship between increasing age and knowledge of diarrhoeal symptoms and a negative relationship between increasing age and knowledge on causes on one hand and increasing age and knowledge on prevention methods on the other. While it is easy to remember “watery stools” as a symptom of diarrhoea through years of experience with the disease, remembering a multiple of causes or preventive measures may not be easy and may require cognitive reasoning. Studies have shown that aging has an important influence on cognitive performance and that factors representing memory and space/reasoning decrease with age [21,22]. 

The random effect variances for symptoms, causes, and overall knowledge are significant at the community level suggesting that there is unmeasured heterogeneity at the level of the community which is not captured by the predictor variables included in the model. This shows that there are substantial within-community clusters on diarrhoea knowledge which are not accounted for by the observed characteristics of responsible mother’s age and education, nearest health facility, existence of an HSA, VHCs or an NGO. This also shows that there are differences in responsible mothers’ knowledge on diarrhoea between communities. In other words, there are hidden and unobserved factors that directly influence responsible mothers’ knowledge on diarrhoea within communities. Such differences may partly be due to different health education messages and/or policies between communities. Morse [2] observed that there were different NGOs operating in Chikhwawa. However, their projects were concentrated in particular areas, hence only benefited those particular communities. Furthermore, different NGOs have different goals in their areas of operation and HSAs are required to carry out work on behalf of the NGOs based on the NGOs’ terms of reference. This means there is no uniformity in the type of projects being executed and health education messages being disseminated. Other reasons may include shared healthcare facilities, cultural, socioeconomic, or environmental experiences not accounted for in the models. Responsible mothers living in the same community, for example, may be more likely to share beliefs on diarrhoea and other diseases through shared experiences. 

## 5. Conclusions

Despite the limitations associated with cross-sectional data, these findings have an important role to play in policy and the promotion of health education messages in Chikhwawa District. While awareness campaigns are conducted in Chikhwawa they are mostly done through hospital personnel [2,23]. The observations in this study show that the involvement of HSAs has a positive impact which can be utilised by increasing the number of and empowering HSAs so that they are equipped and knowledgeable to effectively disseminate messages on diarrhoea and other diseases. It is important to standardise policies in relation to diarrhoea and other diseases. NGOs and all other health promotion players need to coordinate their activities within each district to ensure consistency. HSAs and VHCs must be increased and must cover all communities in Chikhwawa. HSAs and VHCs must be given adequate training to ensure they are able to grasp not only the standards required but also the principles behind them to ensure effective and efficient services to the communities. 
